# 

**Published:** 2020-01-24

**Authors:** Saridogan Ertan

Obstetrics and Gynaecology are gradually becoming two distinct disciplines and many of us are now practicing only one or the other. There are however a number of areas where the gynaecological endoscopic surgeon wanders into the obstetric field. One such example is the use of technology, which is normally used for gynaecological procedures, for the treatment of twin-to-twin transfusion syndrome. In this issue of Facts, Views and Vision, Van der Veeken et al. (page 197) describe the use of fetoscopic laser coagulation of chorionic plate anastomoses for the treatment of twin-to-twin transfusion syndrome in monochorionic twins. The article describes that there is now high quality evidence confirming the efficacy and safety of this technique in increasing fetal survival and reducing paediatric neurological morbidity.

Another feature of the article by Van der Veeken et al. is that it incorporates a number of videos to support the written material. Inclusion of videos will now be a regular feature of Facts, Views and Vision; we have the necessary infrastructure to complement the articles with this visual aid. We will publish ‘video articles’ to describe techniques, instrumentation and interesting cases. The journal of a leading international endoscopic society would be unthinkable without this technology.

Endometriosis is often described as an enigmatic disease. The article by Koninckx et al. (page 209) offers an addition to the existing theories of its etiopathogenesis by exploring the association between genital infections and endometriosis. Contrary to the historical thinking, the uterine, tubal and peritoneal cavities are now thought to have their own microbial communities. The authors identified 31 articles which describe a link between infection and endometriosis. They postulate that alterations in upper genital microbiota could influence genetic/epigenetic events leading to development of endometriosis. They admit that there may be a publication bias in the literature, as studies with a negative outcome are less likely to be published. This review is likely to stimulate further debate in this field and hopefully lead to more structured research on the subject.

The famous pioneer of gynaecological surgery Victor Bonney advised in early 20 th century that the uterine cavity should always be opened during abdominal myomectomy to avoid missing intracavitary fibroids. With availability of high resolution ultrasound and hysteroscopy, identifying submucosal fibroids is no longer a problem and laparoscopic myomectomy is the preferred option when abdominal fibroid removal is needed, except in those with high number of fibroids. Contrary to Bonney’s teaching, we now try to avoid opening the uterine cavity during abdominal myomectomies, presumably to reduce risk of intrauterine adhesion formation. Rajah et al. (page 229) explore the preoperative risk factors that may identify women who are more likely to have uterine cavity breach. They describe that the protrusion ratio, which is described as the proportion of fibroid protruding into the cavity at preoperative ultrasound examination, is the single best predictor of opening the cavity.

The new format Facts, Views and Vision: Journal of the European Society of Gynaecological Endoscopy has already attracted attention from the scientific community. I am confident that the current issue will enhance this interest further and stimulate research, innovation and evidence based practice in our field.

Ertan Saridogan, ESGE Editor

